# Spectroscopic single-molecule localization microscopy: applications and prospective

**DOI:** 10.1186/s40580-023-00363-9

**Published:** 2023-03-21

**Authors:** Benjamin Brenner, Cheng Sun, Françisco M. Raymo, Hao F. Zhang

**Affiliations:** 1 Department of Biomedical Engineering, Northwestern University, Evanston, IL 60208 USA; 2 Department of Mechanical Engineering, Northwestern University, Evanston, IL 60208 USA; 3 Department of Chemistry, University of Miami, Coral Gables, FL 33146 USA

**Keywords:** Super-resolution microscopy, Single-molecule localization microscopy, Spectroscopy, Polarity sensing, Single particle tracking

## Abstract

Single-molecule localization microscopy (SMLM) breaks the optical diffraction limit by numerically localizing sparse fluorescence emitters to achieve super-resolution imaging. Spectroscopic SMLM or sSMLM further allows simultaneous spectroscopy and super-resolution imaging of fluorescence molecules. Hence, sSMLM can extract spectral features with single-molecule sensitivity, higher precision, and higher multiplexity than traditional multicolor microscopy modalities. These new capabilities enabled advanced multiplexed and functional cellular imaging applications. While sSMLM suffers from reduced spatial precision compared to conventional SMLM due to splitting photons to form spatial and spectral images, several methods have been reported to mitigate these weaknesses through innovative optical design and image processing techniques. This review summarizes the recent progress in sSMLM, its applications, and our perspective on future work.

**Graphical Abstract:**

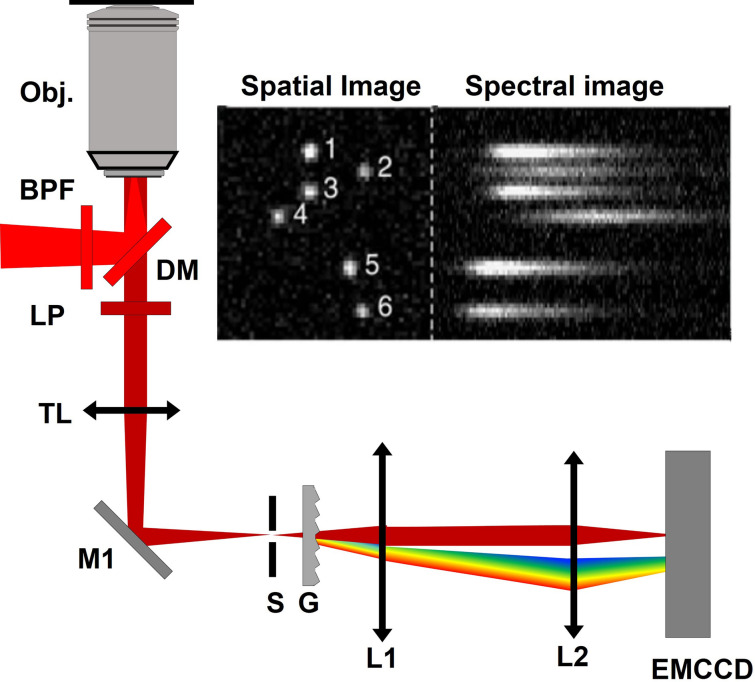

## Introduction

The resolution of light microscopy has long been constrained by the optical diffraction limit [1]. The diffraction limit states that two objects cannot be distinguished from each other using a light microscope if they are separated by less than roughly half the wavelength of the probing light. This limit is the consequence of the optical point spread function (PSF), the diffraction pattern formed by light from a point source when a lens collects it and focuses on a detector. In a standard light microscope, the PSF is shaped as an airy disc with its full-width-at-half-maximum (FWHM) defined by the Abbe limit

1 $$\mathrm{FWHM}=\frac{\lambda }{2n*\mathit{sin}\left(\theta \right)},$$

where $\lambda$ [nm] is the wavelength of the detected light; $n$ [dimensionless] is the refractive index of the medium through which an object is imaged; and $\theta$ [°] is the collection angle of the lens. An image acquired by a diffraction-limited microscope may be considered the ground truth convolved with the PSF of the optical system. As an Airy disc somewhat resembles a 2D Gaussian function, diffraction-limited imaging can be treated as blurring the true image by a Gaussian kernel. Super-resolution microscopy describes any light microscopy technique that breaks the diffraction limit. In practice, it generally applies to fluorescence microscopy [2], although researchers have reported several label-free super-resolution microscopy technologies [3–6].

Structured illumination microscopy (SIM) achieves super-resolution by illuminating a sample with patterned light in various orientations to extend the sampled Fourier space with higher spatial frequency components [7]. Stimulated emission depletion (STED) microscopy achieves super-resolution by illuminating the sample with an excitation beam and a donut-shaped depletion beam, resulting in an effective focal spot with a diameter below the diffraction limit [8, 9].

Single-molecule localization microscopy (SMLM) describes a group of super-resolution techniques that improve imaging resolution by sparsely sampling fluorescent labels. Stochastic optical reconstruction microscopy (STORM) uses fluorophores that undergo photoswitching (or blinking) when imaged under high-intensity light in an oxygen-scavenging buffer [10]. In each frame of a STORM image, only a sparse subset of fluorophores emits light, forming several distinct PSFs on the detector. These PSFs are generally approximated by a 2D Gaussian function, whose peak is considered the true location of the fluorophore. An algorithm goes through each frame of STORM data and fits a 2D Gaussian function to each PSF in every frame using a fitting regime, such as maximum likelihood estimation (MLE), to estimate the location of each fluorophore with sub-diffraction limit precision [11]. The algorithm then visualizes all the localized fluorophores in a 2D [10, 12] or 3D [13, 14] histogram.

Researchers demonstrated multicolor SMLM using one or more dichroic mirrors in the detection path to separate the emission photons into different color channels [15], using sequential activation pulses of different wavelengths [16, 17], or using sequential labeling [18]. However, these methods are analogous to diffraction-limited multicolor microscopy [19–21] and do not take full advantage of the single-molecule sensitivity enabled by SMLM. Broeken et al. used a spatial light modulator (SLM) to engineer a Gaussian PSF with spectra diffracted on either side [22]. They subsequently demonstrated that they could encode axial information in the orientation of the spectra [23]. However, introducing an SLM led to significant photon loss, and the spectral features were compressed into a small space near the PSF center, limiting spectral precision.

**Fig. 1:**
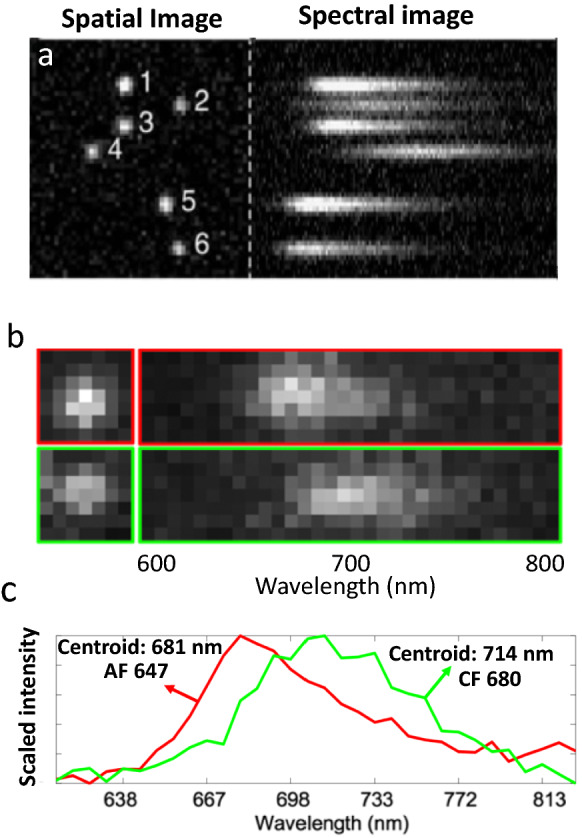
**a** Example of spatial (left) and spectral (right) images in sSMLM; **b** Spatial and spectral images of AF 647 (top, red) and CF 680 (top, green); **c** Calibrated spectra of imaged AF647 and CF 680 (Reprinted with permission from [25] and [31])

**Fig. 2:**
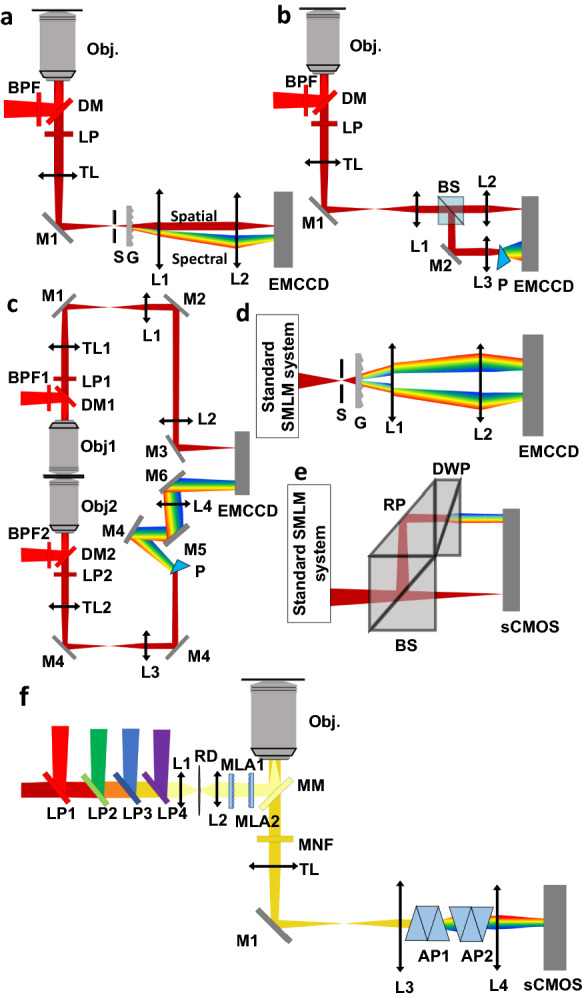
**a** Schematic of single-objective, grating-based sSMLM. Obj.: objective lens; DM: dichroic mirror; BPF: band-pass filter; LP: long-pass filter; TL: tube lens; M: mirror; S: slit; G: diffraction grating; L: lens; **b** Schematic of single-objective prism-based sSMLM. BS; beam-splitter; P: prism; **c** Schematic of dual-objective, prism-based sSMLM; **d** Schematic of symmetrically dispersed sSMLM; **e** Schematic of DWP-based sSMLM. RP: right prism; **f** Schematic of amici prism-based sSMLM. RD: rotating diffuser; MLA: microlens array; MM: multichroic mirror; MNF: multinotch filter; AP: Amici prism

Between 2015 and 2016, several groups demonstrated spectroscopic SMLM (sSMLM), which refracts each single-molecule image using a dispersive element to simultaneously capture the locations of single-molecule emissions and their spectra [24–27]. In sSMLM, each frame contains two simultaneously acquired images: a spatial image containing molecules’ PSFs, and a spectral image containing their corresponding spectra (Fig. 1a). The spectral image can reveal the species of each fluorescent molecule based on its spectral position and shape (Fig. 1b, c). Researchers have implemented sSMLM in several ways, such as using a diffraction grating (Fig. 2a), a beam splitter and a prism (Fig. 2b), or using two objectives with a prism (Fig. 2c). In this review, we summarize different configurations of sSMLM and discuss their respective strengths and weaknesses. We also discuss the applications of sSMLM in multicolor cellular imaging, polarity-sensing, and chemical conformation detection. Then we discuss image processing techniques to enhance sSMLM, including machine learning. Lastly, we discuss future directions of sSMLM with a particular focus on multicolor single particle tracking (SPT).

## Implementations of sSMLM

In 2016, our group introduced the first grating-based sSMLM [25] (Fig. 2a), using the zeroth-order beam for spatial imaging and the first-order beam for spectral imaging. One drawback of grating-based sSMLM was limited efficiency. As we reported previously, 49% of the emitted photons went to the zeroth-order beam, 21% went to the first-order beam, and 30% of the photons were attenuated by the grating and were not used for image reconstruction [28]. Losing photons in sSMLM led to reduced spatial and spectral precisions. Another drawback of this implementation was that the grating’s diffraction angle and the field of view (FOV) were inherently coupled. When reducing the desired spectral dispersion, the smaller diffraction angle also reduced FOV.

Moon et al. introduced single-objective prism-based sSMLM (Fig. 2b) [28], which used a beam splitter (BS) first to split the emitted photons into two beams, one for spatial imaging and one, after passing through a prism, for spectral imaging. While this method avoided photon loss induced by the grating, it still suffered from a substantial photon loss caused by the much-increased number of air-glass interfaces than grating-based sSMLM. Unlike grating-based sSMLM, single-objective prism-based sSMLM requires a BS and an additional mirror in the detection path. For example, if we assume a 30:70 splitting ratio by the BS, the spectral beam efficiency is comparable to, and the spatial beam efficiency is slightly higher than the grating-based sSMLM using the same splitting ratio. However, the grating-based sSMLM used only a single component, yielding a much simpler optical alignment and maintenance. In addition, grating-based sSMLM provides a linear spectral dispersion, making calibration and image processing more straightforward.

Zhang et al. introduced a dual-objective prism-based sSMLM system (Fig. 2c) [24], where they used two objective lenses, one for spatial and one for spectral imaging. This 4-pi detection [29] collected twice as many photons as sSMLM using a single objective lens, leading to much improved spatial and spectral precisons. However, 4-pi systems are far more challenging to align and maintain and require a specialized optical setup, unlike the other two that are mostly compitable with standard SMLM systems.

Since these initial reports, several groups introduced modified sSMLM implementations. Attempting to improve the spatial and spectral precisions of the grating-based sSMLM system, Song et al. introduced symmetrically dispersed sSMLM (SDsSMLM) (Fig. 2d) [30]. Instead of having one spatial and one spectral beam, they used a grating to generate equally dispersed -1st and 1st orders resulting in two mirrored spectral images. The authors estimated the middle point of the centroids of each pair of spectral images to generate a virtual spatial image. The authors further combined the two spectral images with respect to the middle point for spectral analysis. This way, all collected photons collectively contributed to spatial localization and spectral analysis, improving both spatial and spectral precisions. However, it is challenging to fabricate a grating with minimized zeroth-order and higher-order diffractions and a transmission loss of less than 15%.

Recently, Song et al. introduced a dual-wedge prism (DWP)-based sSMLM. In DWP-based sSMLM, the authors assembled all optical components into a single-objective prism-based system that combined the advantage of the low transmission loss in prism-based sSMLM and the compactness of grating-based sSMLM in a single, compact module, which can be easily inserted into the imaging path (Fig. 2e) [31]. The optical assembly included a beam splitter, a prism, and two wedge prisms. The photons passing through the beam splitter formed a spatial image, and the photons reflected by the beam splitter and the prism formed the spectral image after being dispersed by the two wedge prisms. As a result, DWP-based sSMLM did not suffer from the high transmission loss caused by the grating and minimized the number of air-glass interfaces compared to existing prism-based sSMLM to achieve the highest photon utilization. In addition, unlike in grating-based sSMLM [25], the FOV of DWP-based sSMLM is independent of spectral dispersion [31].

Jeffet et al. introduced an Amici prism-based sSMLM system [32], which allows the user to tune spectral dispersion by rotating one of the prisms (Fig. 2f). One drawback of this implementation is that spatial and spectral imaging must be done sequentially. Users need to orient the prisms to achieve zero spectral dispersion for spatial imaging and then reorient them to achieve the desired spectral dispersion for spectral imaging. This weakness limits its applications to parallel particle tracking, where the particle locations change temporally, but spectra are relatively stable.

## Multiplexed imaging using sSMLM

**Fig. 3:**
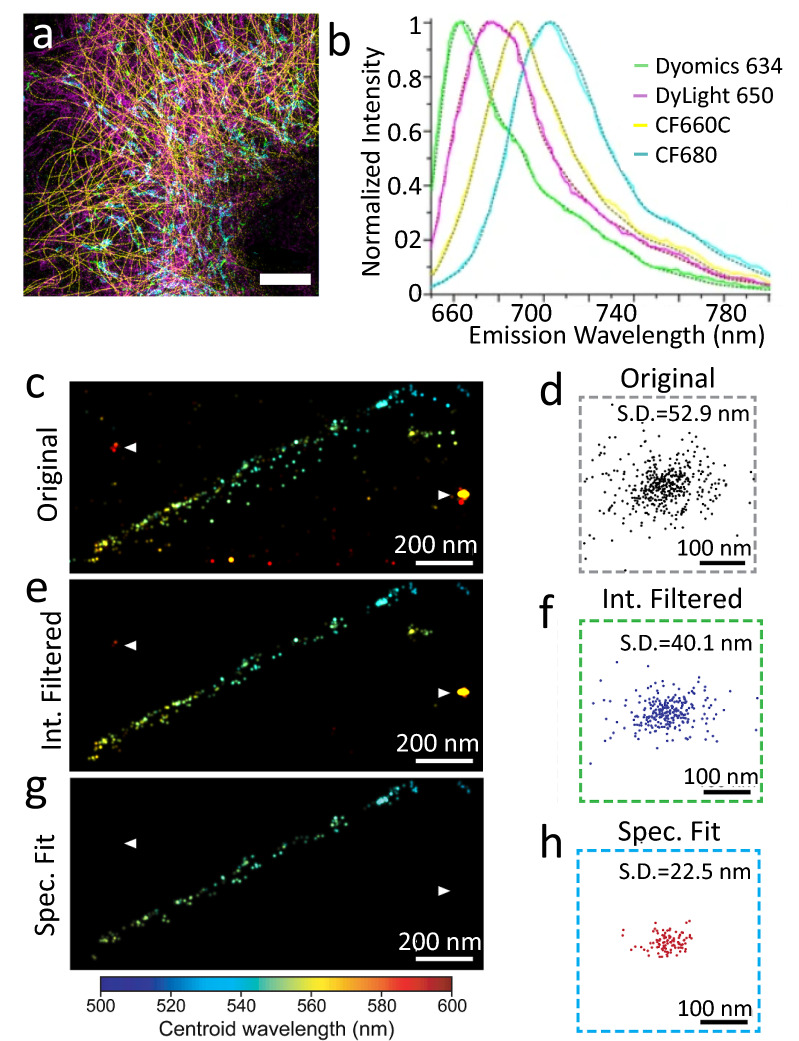
**a** Multicolor cell image of peroxisome, vimentin, micro-tubulin, and mitochondria. Scale bar: 2 μm; **b** Emission spectra of Dy634, DL650, CF660, and CF680. Reprinted from [33] and [24] with permission; **c**, **d** Unfiltered image of stretched lambda phage DNA and the standard deviation from the expected ground truth; **e**, **f** Intensity-filtered image of stretched lambda phage DNA and the standard deviation from the expected ground truth; **g**, **h** Spectrally-filtered image of stretched lambda phage DNA and the standard deviation from the expected ground truth (Reprinted with permission from [34])

Early demonstrations of sSMLM largely focused on multicolor cellular imaging [24, 33]. In particular, multicolor studies emphasized that sSMLM made it possible to pair fluorophores with similar spectral signatures, such as AF647 and AF660, with low cross-talk, due to its high spectral sensitivity [33]. Zhang et al. [24] demonstrated 4-color sSMLM cell imaging using fluorophores with highly overlapped emission spectra between 660 and 710 nm (Fig. 3a, b). Having several fluorophores within the same spectral range makes it possible to perform simultaneous multicolor imaging using a single excitation wavelength and a single filter-set, considerably simplifying the optical setup. Zhang et al. [33] performed multicolor imaging using CF660 and AF647, and highlighted the importance of balancing the level of spectral dispersion with the intensity of emissions in order to maximize spectral precision and thereby minimize cross-talk.

Davis et al. [34] used sSMLM to differentiate between desired signals and fluorescent impurities in biological samples [34]. They imaged unstained cover glasses and cells with and without poly-l-Lysine treatment and characterized their spectral and intensity characteristics, which they found were distinct from the desired signal. They then imaged a stretched lambda phage DNA sample labeled with YOYO. They compared the sSMLM image to the expected ground truth (a straight line) after applying various filtering strategies to generate an optimized filtering algorithm for impurity removal. They found that unfiltered and intensity-filtered images (Fig. 3c–f) had a wider spread of localizations around the stretched DNA than the spectrum-filtered image (Fig. 3g, h), indicating that spectrum-filtering was more successful at removing influence from fluorescence impurities.

## Polarity sensing using sSMLM and Nile red

Using the polarity-reporter dye Nile red (NR), which changes its emission spectrum in responding to the polarity of the surrounding environment, sSMLM can detect subtle changes in local polarity intracellularly. Similar to other benzophenoxazine dyes, NR is lipophilic [35] and is often used to label both external and internal cellular lipid membranes [27]. The sensitivity of NR to the polarity of the surrounding environment is a consequence of the significant difference between the dipole moments of its ground and excited states. Specifically, the dipole moment of NR increases from *ca.* 9 to 14 D with excitation [36]. As a result, polar media stabilize the excited state, relative to the ground state, decreasing the energy gap associated with the radiative decay from the former to the latter state. The overall result is a red shift in emission with an increase in solvent polarity. NR’s emission dynamics and reversible binding to lipids made it compatible with SMLM and point accumulation in nanoscopic topography (PAINT) [37].

**Fig. 4:**
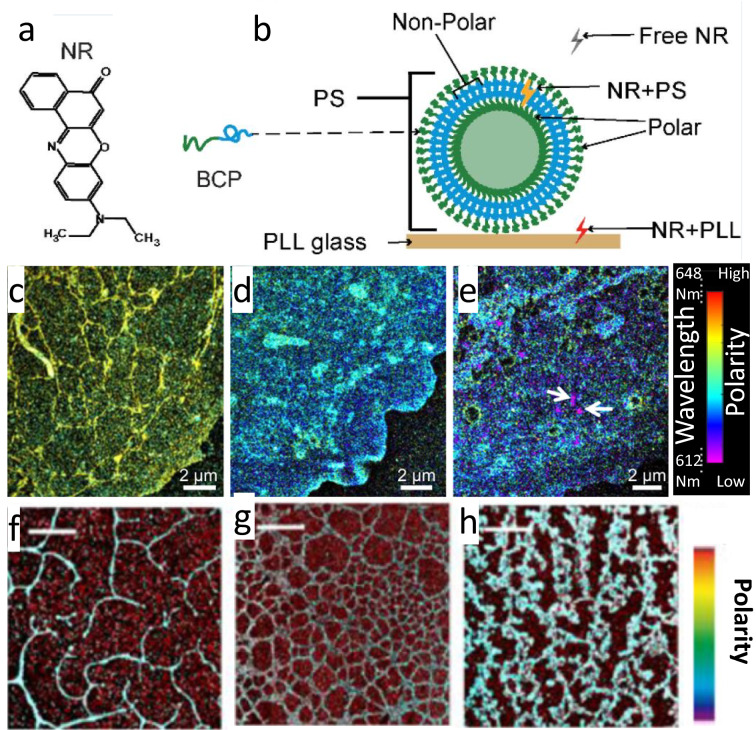
**a** Chemical structure of NR; **b** Illustration of self-assembled polymersome showing that NR emits light at different wavelengths depending on whether it is free-floating, bound to the PLL-coated cover-glass, or bound to the polymersome. Reprinted with permission from [35]. Typical sSMLM images of fixed COS-7 cell membranes labeled with NR after **c** cholesterol depletion with MβCD, **d** mild cholesterol enrichment with 1 mM water-soluble cholesterol, and **e** cholesterol enrichment with 5 mM water-soluble cholesterol. Reprinted with permission from [28]. Typical sSMLM images of **f** 5:1, **g** 1:1, and **h** 1:5 mixes of PVC/PIB polymer blends. Scale bars: 5 μm (Reprinted with permission from [41])

Davis et al. [35] demonstrated that NR could label polymersome nanocarriers that reversibly self-assemble from block copolymers. Polymersomes nanocarriers have the potential to deliver drugs or vaccines into intracellular space. The structure of polymersomes is analogous to a lipid bilayer, with a non-polar segment insulated on both ends from the aqueous solution with a polar segment. Davis et al. showed that NR reversibly binds to the non-polar segment of polymersomes, enabling sSMLM to visualize the polymersomes at nano-scale resolution. They also showed that because the free-floating NR and NR bound to the PLL-coated cover glass experienced a different local polarity than NR bound to polymersomes, sSMLM detected the polarity-induced emission spectral shift (Fig. 4b) to reject unbounded NR signals.

Moon et al. imaged NR using sSMLM to classify the properties of cellular lipid membranes [28]. In this study, PtK2 cell plasma membranes were labeled with NR after being treated with different concentrations of MβCB to deplete cholesterol or water-soluble cholesterol to enrich cholesterol (Fig. 4c–e). The authors showed that cholesterol depletion was significantly correlated with lower polarity based on the solvatochromic properties of NR detected by sSMLM [28]. They also detected low-polarity, high-cholesterol rafts in the plasma membrane [28]. Danylchuk et al. demonstrated a modified NR specific to plasma membranes as opposed to all cellular lipids with enhanced photoswitching properties [38]. They introduced a new membrane-targeting moiety based on an anionic sulfonate head group and an alipophilicalkyl chain. The authors then used sSMLM to map lipid orders on the plasma membrane of live COS-7 cells [38]. Yan et al. performed sSMLM on COS-7 lipid membranes concurrently labeled with NR and BDP-TMR alkyne, both lipophilic fluorophores, to measure polarity and diffusivity, respectively [39, 40]. The authors used the NR spectrum to measure polarity and lipid order [38], and used 1-ms excitation pulses at 560 nm to briefly excite BDP-TMR alkyne and measure displacement to calculate local diffusivity. The authors found that while diffusivity was generally correlated with lipid order, there were exceptions, such as endoplasmic reticulum peroxisome contact sites [39].

Several groups applied sSMLM and NR to classify materials’ surface properties. For example, Park et al. characterized the nanoscopic properties, in particular, polarity, of polymer materials [41]. They spin-coated mixed polymer and NR solutions onto glass coverslips and used sSMLM to map the polarity of different polymer materials. They found that polymer materials with a higher ratio blend of PIB to PVC exhibited more low-polarity islands (Fig. 4f–i). Xiang et al. [42] and Kim et al. [43] investigated the polarity of adlayers using sSMLM and NR. Xiang et al. immersed the coverslips in various organic solutions to form adlayers on coverslips. After adlayer formation, the authors immersed the coverslips in an aqueous NR solution for sSMLM imaging. They found that adlayers formed from more polar solvents caused greater redshifts in NR’s emission spectrum and visualized the adlayers’ structures [42].

## Detecting chemical properties of fluorescent molecules using sSMLM

**Fig. 5:**
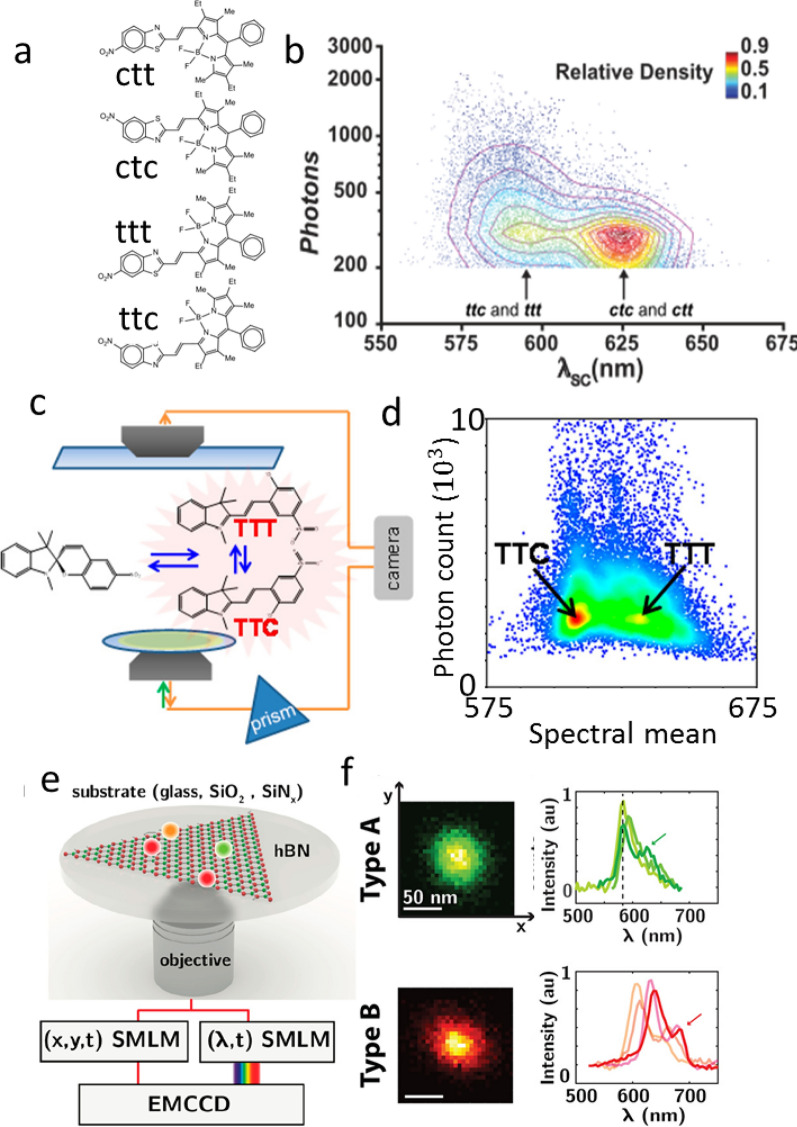
**a** Structures of the four conformational isomers of a BODIPY derivative; **b** Scatterplot of the corresponding single-molecule emission intensity against the spectral centroid with the relative density of distribution; **c** Schematic of the optical configuration to detect the two isomers of merocyanine; **d** Spectral histogram showing the detection of two distinct populations of merocyanine. Reprinted with permission from [47]; **e** Illustration of the sSMLM imaging of emitters on hexagonal boron nitride; **f** Spatial image and spectrum of the two defects on hexagonal boron nitride (Reprinted with permission from [50])

When a mixture contains several isomers of a fluorescent molecule, each with a unique spectral signature, the spatial distribution of isomers is of great interest [44, 45]. However, standard multicolor imaging techniques either lack the sensitivity to differentiate spectral alterations or the sensitivity for spatial mapping at the single-molecule level. Thus, sSMLM can be an ideal tool to satisfy both the spectral and spatial sensitivity needs. Sansalone et al. [46] applied sSMLM to differentiate the conformational isomers of three BODIPY derivatives. The four co-existing conformational isomers (Fig. 5a) of each compound have all extended electronic delocalization, but differ in the relative orientation of the heterocyclic fragments at the termini of the ethyne bridge. Consistently, sSMLM reveals two main populations of emissive molecules with spectral-centroid distributions (Fig. 5b) centered at 593 and 623 nm respectively. The former population was assigned to the two conformational isomers (*ttc* and *ttt*) differing only in the relative orientation of the BODIPY chromophore, with the aid of time-dependent density functional theory (TDDFT) calculations. The latter population was assigned instead to the two conformational isomers (*ctt* and *ctt*) differing in the orientation of the benzothiazole heterocycle, again on the basis of TDDFT calculations.

Kim et al. applied sSMLM to study the isomerization of the nonfluorescent molecule spiropyran into the fluorescent merocyanine (Fig. 5c) [47]. The authors found two populations of spectra (Fig. 5d), which they conjectured corresponded to two conformational isomers (TTT and TTC) of the merocyanine form. Similarly, Zhang et al. used sSMLM to examine spectral heterogeneity in Rhodamine B, a fluorophore exhibiting large spectral heterogeneity [48]. They compared the spectral heterogeneity of Rhodamine B (RhB), which has a flexible *N*,*N*-dialkylamino group, to that of Rhodamine 101 (Rh101), which is structurally similar but with a rigid *N*,*N*-dialkylamino group. They found that Rh101 had lower heterogeneity, indicating that spectral heterogeneity in RhB could be caused by conformational changes of the *N*,*N*-dialkylamino group.

Researchers also applied sSMLM to characterize unlabeled autofluorescent surfaces [49, 50], such as defects in hexagonal boron nitride (hBN) layers (Fig. 5e). Chemical vapor deposition of hBN can be an excellent insulating layer in nanoelectronics; however, any defects can be detrimental to its electrical properties [51]. These sub-diffractional defects are hypothesized to be either nitrogen or boron vacancies, which Stern et al. observed emitting photons with a narrow wavelength range using sSMLM [49]. Stern et al. further distinguished two species of defects (Fig. 5f) based on the detected emission spectral signatures. Although electron microscopic technologies provide sufficient spatial resolution to resolve these defects, they cannot recognize the species of the defects, and their laborious sample preparation process often introduces new defects. However, further investigation is required to thoroughly understand the atomic structure of these defects [50].

## Quantitative imaging and machine-learning-based analysis using sSMLM

The resolution of SMLM ultimately depends on how precisely a 2D Gaussian function can be fitted to each PSF. In 2014, Rieger et al. published their seminal work [11] and described how localization precision could be estimated based on parameters of the fitted Gaussian, such as photon count and signal spreading [11]. The authors found that MLE fitting achieved the Cramer Rao lower bound (CRLB), meaning it is the best possible fitting method, assuming that a PSF can be approximated by a Gaussian distribution. Rieger et al. defined localization precision as

2 $$\Delta {x}^{2}=\frac{{F}_{EM}{\sigma }^{2}+\frac{{a}^{2}}{12}}{N}\left(1+4\tau +\sqrt{\frac{2\tau }{1+4\tau }}\right),$$

where

3 $$\tau =\frac{2\pi b\left({\sigma }^{2}+\frac{{a}^{2}}{12}\right)}{N{a}^{2}};$$

${F}_{EM}$ [photons] is the gain noise from an EMCCD camera; *N* [photons] is photon count per PSF; $\sigma$ [nm] is the standard deviation of the fitted Gaussian function; *a* [nm] is pixel size; and *b* [photons] is background photons per pixel. Since parameters in this equation are estimated from the fitted Gaussian distribution, they each have their own uncertainties [11].

Spectral precision follows a similar principle; however, additional parameters, such as spectral dispersion that causes additional signal spreading, must be considered. Song et al. [52] defined spectral precision as

4 $${\sigma }_{\lambda }^{2}={F}_{EM}^{2}\left(\frac{{s}_{\lambda }^{2}}{N}+\frac{16B{s}_{\lambda }^{2}}{3{N}^{2}}\right)+\frac{1024{n}_{ro}^{2}{s}_{\lambda }^{3}{s}_{y}}{3\Delta \lambda \Delta y{N}^{2}}+\frac{\Delta {\lambda }^{2}}{12},$$

where ${s}_{\lambda }$[nm] is the standard deviation of the spectral PSF in the direction of spectral dispersion; ${s}_{y}$ [pixels] is the spread of the spectral PSF in the non-dispersed direction; ${n}_{ro}$ [photons] is readout noise per pixel; $\Delta \lambda$ [nm/pixel] is the spectral dispersion; *B* [photons] is background noise per pixel; $\Delta y$ [nm/pixel] is the pixel size in the spatial domain. Based on this definition, Song et al. identified the ideal spectral dispersion for a given experimental setup and the expected signal-to-noise ratio (SNR). This ideal spectral dispersion is a trade-off between the error from signal spreading caused by wide spectral dispersion and the error from spectral shift caused by a narrow dispersion. However, this uncertainty equation only applies to the spectral centroid as calculated using the weighted mean centroid method reported by Zhang et al. and Song et al. [24, 52]. Meanwhile, other groups have reported using Gaussian fitting to identify the spectral centroid [27]. However, no studies have quantitatively compared these two methods. Martens et al. reported a higher emitter density, higher spectral SNR, and better temporal resolution than other sSMLM implementations using a narrow spectral dispersion; however, this may introduce a higher spectral shift error [53].

**Fig. 6:**
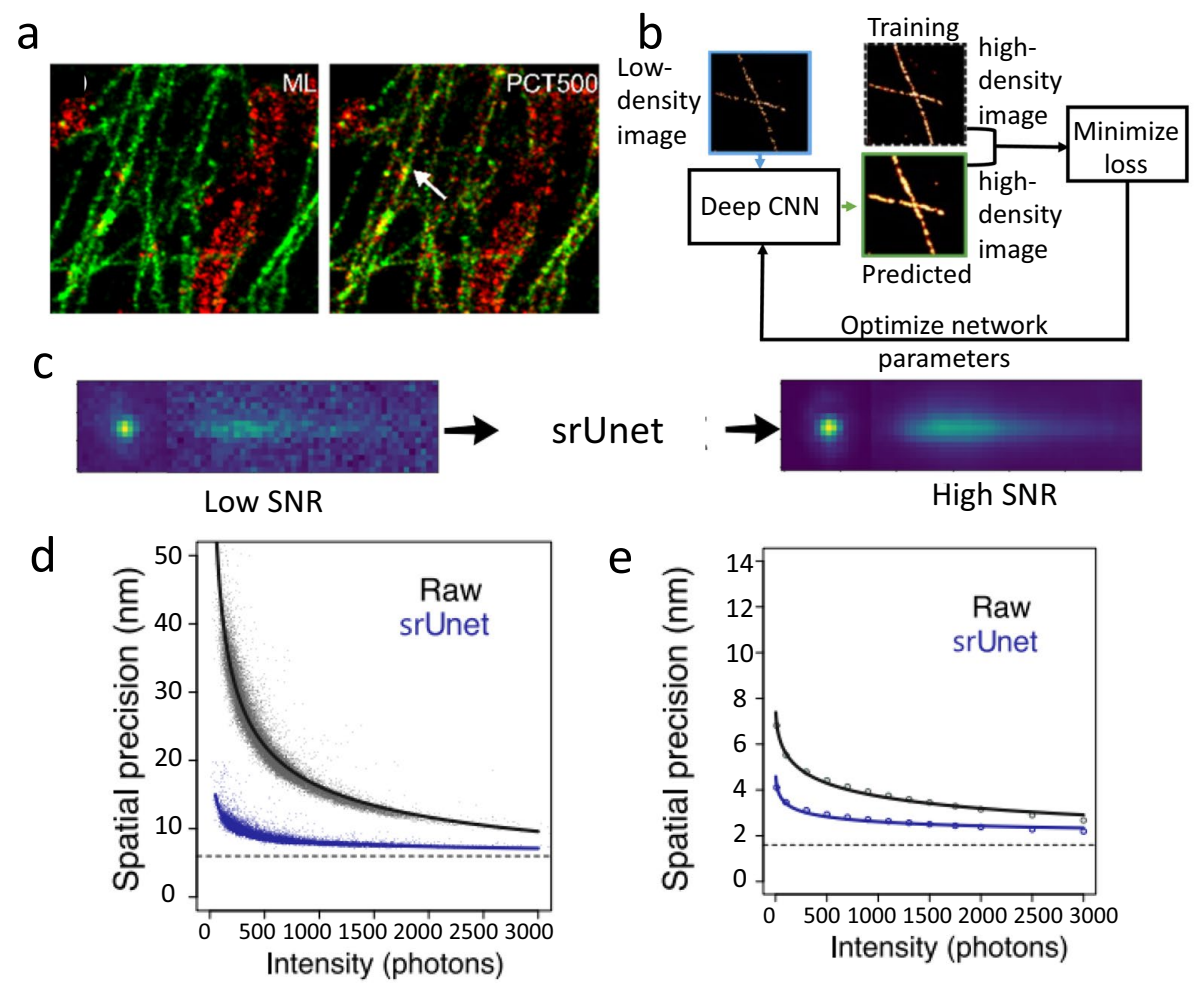
**a** Comparing machine-learning-based and spectral centroid-based classification in sSMLM. Reprinted with permission from [54]; **b** Flowchart showing the training of srUnet to produce estimated high-density images Reprinted with permission from [55]. **c**–**e** Improvement in raw image quality of spatial and spectral image, spatial precision, and spectral precision by srUnet (Reprinted with permission from [56])

Several studies attempted to improve spatial and/or spectral precision in sSMLM using machine learning. Zhang et al. [54] reported a neural network-based spectral classification in multiplexed sSMLM imaging, reducing the cross-talk among different spectral bands compared to the weighted spectral centroid (Fig. 6a). Because sSMLM splits emitted photons into spatial and spectral images, a higher percentage of spatial localizations must be discarded in sSMLM than in SMLM to maintain a comparable spatial precision. Furthermore, due to signal spreading along the spectral dispersion axis in the spectral image, a greater sparsity is also required in sSMLM. Hence, sSMLM usually needs more frames and longer acquisition time than traditional SMLM. Gaire et al. developed a machine-learning method to address this limitation [55]. They acquired high-density sSMLM datasets, then generated a low-density image from a fraction of the acquired datasets. They first trained a neural network using low-density images and optimized it to generate output resembling the high-density images (Fig. 6b). Then, they used the network to predict high-density images from low-density acquisition, reducing the data acquisition time. Manko et al. developed spectrally-resolved U-net (srUnet), a neural network to improve the SNR from noisy spatial and spectral PSFs (Fig. 6c), and showed improved spatial and spectral precisions after SNR improvement [56] (Fig. 6d, e).

## Single particle tracking using sSMLM

**Fig. 7:**
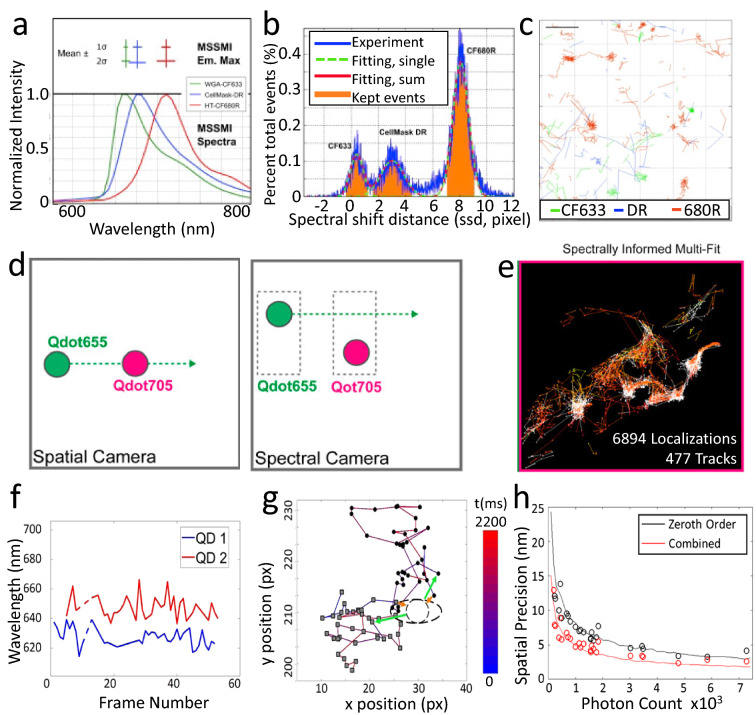
**a** Average emission spectra of CF 633, CellMask DR, and CF680R; **b** Histogram of identified spectral centroids showing each fluorophore species resolved into a separated spectral peak. **c** Multiplexed SPT in U2OS cells. Reprinted with permission from [60]; **d** Illustration of sSMLM tracking two Qdot species; **e** Trajectories for two-color QD tracking with spectrally informed trajectory assignments. Reprinted with permission from [61]. **h** improved spatial precision by integrating spatial information from the spectral image in the tracking trajectory; **f**, **g** spectral tagging of individual quantum dots to separate trajectories with spatial overlapping (Reprinted with permission from [63])

Single particle tracking (SPT) has long been associated with SMLM [57–59]. When tracking a fluorescent particle, Gaussian fitting of the imaged particle could identify the particle’s location with super-resolution precision when the particle size is below the optical diffraction limit. Huang et al. demonstrated 3-color SPT on the plasma membrane using sSMLM in live U2OS cells [60]. They used CF633, CellMask DR (peak absorbance 669 nm), and CF680R (Fig. 7a, b), conjugated to WGA (a probe that targets glycoproteins), a membrane anchor, and HT (a probe that targets transferrin receptors). The authors first attached the fluorophores to the plasma membrane and tracked them diffusing across the membrane simultaneously (Fig. 7c).

Butler et al. [61] and Dong et al. [62] showed 3D spectroscopic SPT of quantum dots (QDs) using sSMLM (Fig. 7d, e) [61, 62]. Based on spatial information only, traditional SPT cannot differentiate two particles if they happen to overlap spatially within one or more frames, and there is no method to confidently assign their post-crossing trajectories to their corresponding pre-crossing trajectories [59]. Brenner et al. [63] developed a tracking algorithm designed explicitly for spectroscopic SPT. They used the spectrum to tag each particle so that the post-crossing trajectories could be assigned to their respective pre-crossing trajectories based on the particles’ spectral centroids. Because of the high sensitivity of sSMLM and the high spectral heterogeneity of QDs, this spectral-tagged SPT worked when both QDs were of the same species (Fig. 7f, g). Furthermore, they showed that combining spectral tagging with the trajectory constructed from the spatial locations improved the tracking precision (Fig. 7h, i) [63].

## Prospectives of spectroscopic SPT using sSMLM

Although several groups demonstrated the feasibility of spectroscopic SPT using sSMLM in various samples, there has been a lack of practical studies to investigate biological questions. Here, we provide our perspective on future applications of spectroscopic SPT to biological research.

One potential application of spectroscopic SPT is elucidating the signaling pathways that occur as T-cells become activated and bind to their targets via T-cell receptors (TCR). This topic is particularly interesting to clinical scientists exploiting the mechanism to generate chimeric antigen receptor (CAR) t-cells, which can target cancer cells and are used in cancer therapy [64]. Ito et al. used simultaneous 3-color diffraction-limited imaging and single-color SPT to elucidate the TCR activation process [65]. The authors labeled CD45, CD3ζ, and CD3ε (molecules involved in T-cell activation) in immobilized Jurkat T-cells and imaged them. They then sequentially tracked CD3ε and CD45 and found two diffusion states (fast and slow) existed within TCR microclusters. This study could be improved by spectroscopic SPT using sSMLM to determine whether these diffusion states are spatially correlated with TCR and linker for activation of T-cells (LAT) protein domains, which are only detectable using super-resolution imaging [66]. Another study showed that upon the formation of a synapse between a CAR-TCR and its target, cytotoxic granules converge at the microtubule organizing center and are later expelled via exocytosis into the target cell [67]. Applying multiplexed spectroscopic SPT could determine whether the granules are being transported by the microtubule or converging from cytokine signaling and could simultaneously track other proteins, such as phosphorylated lymphocyte-specific protein tyrosine kinase (phospho-Lck), essential to synapse formation [68]. These proteins’ trajectories could be further determined with super-resolution with the TCR locations.

Moon et al. tracked single mRNAs labeled with a fluorophore-attached HALO tag as the mRNAs moved between ribonucleoprotein granules (clusters of proteins and RNA that form when the cell is stressed) and P-bodies [69]. They found that, as opposed to translating mRNAs, non-translating mRNAs formed more long-lasting interactions with the granules, indicating that the untranslated region of mRNA formed higher-valency interactions with the ribonucleoprotein granules. Such a higher-valency interaction may be a mechanism for protecting untranslated mRNA when the cell is stressed. A follow-up study using sSMLM can perform spectroscopic SPT to track specific mRNA sequences that are either repressed or upregulated in response to stress.

Another group used SPT to show the intake of HIV-1 into cells via endocytosis [70]. The authors postulate that HIV-1 may rely on unidentified endosomal factors to undergo fusion with the cellular plasma membrane. Using sSMLM, researchers may perform spectroscopic SPT to simultaneously track several endosomal factors and HIV-1 as it fuses with a cell. The endosomal factors involved in HIV-1 fusion could potentially be identified based on imaged proximity and length of interaction. These endosomal factors could then be targets in HIV-1 therapies.

## Discussion and conclusion

Despite the strengths mentioned above, sSMLM also has several weaknesses. First, sSMLM suffers from a limited temporal resolution. The sparsity requirement for spatial localization means that a large number of frames are necessary to generate an image that is well-populated with localizations. As a result, sSMLM’s applications to live cells are limited, especially when a temporal resolution higher than one minute is required [71]. A few groups attempted to improve the temporal resolution by introducing multi-emitter fitting algorithms, which relax the sparsity constraint by fitting overlapping PSFs [71–73] and demonstrated SMLM imaging of relatively fast-paced events in live cells [71]. To our knowledge, however, no groups attempted to implement this algorithm for sSMLM. One challenge is that in the spectral images acquired by sSMLM, each fluorophore species has a unique spectral shape, making it challenging to apply a universal PSF template to fit overlapping PSFs. This challenge is further complicated by spectral heterogeneity, where each fluorophore has a unique spectral signature, even if they are of the same species [28, 48]. One solution to this challenge could be adapting super-resolution optical fluctuation imaging (SOFI), which does not rely on any PSF template, but instead adjusts the intensity of each pixel according to its level of intensity auto-correlation throughout the acquisition [74]. However, while SOFI results in super-resolution images, it does not localize each molecule individually. Therefore, if implemented in sSMLM, there would be a loss in single-molecule sensitivity, which has been a major benefit of sSMLM [35, 47, 75].

Second, the methods to identify the spectral centroid in sSMLM image reconstruction are currently fragmented, either using Gaussian fitting [27] or the weighted mean [24, 52]. It is unclear which method yields higher spectral precision. While Song et al. [52] derived an analytical expression for the spectral precision using the weighted mean method, there has been no study to compare it with Gaussian fitting. One potential concern about the Gaussian fitting method is that a fluorophore’s emission spectrum may not resemble a Gaussian profile as much as a spatial PSF. A library containing all commonly used single-molecule emission spectra in SMLM, which establishes the gold standard for spectral fitting, could benefit the broader sSMLM field.

In addition, existing molecule labels are primarily designed for traditional SMLM, where the maximum number of multiple labels is usually four or fewer [24, 31, 33, 54]. Both multiplexed super-resolution and spectroscopic SPT can significantly benefit from specially designed fluorophores that enable highly multiplexed (> 10) molecular labeling. Moreover, new fluorophores that alter emission spectra responding to more local environmental parameters will enable a broader range of functional imaging beyond polarity sensing.

In conclusion, the utility of sSMLM has already been proven in a remarkable breadth of biological and chemical applications, including multicolor cellular imaging [24, 33], polarity sensing [27, 28, 35, 38], chemical characterization [46–48], and multiplexed SPT [60–62]. However, due to a lack of standardization in optics [24, 28, 31] and image processing techniques [25, 27, 52], reduced spatial precision compared to SMLM [25, 28, 30, 55], and a lack of spectrally optimal fluorescent labels [76], the technology has yet to achieve its full potential.

## Data Availability

Not applicable.
